# Economies as 'Makers' or 'Users': Rectifying the Polysemic Quandary with a Dualist Taxonomy

**DOI:** 10.1007/s13132-023-01247-3

**Published:** 2023-03-21

**Authors:** Vic Benuyenah

**Affiliations:** Birmingham Business School, University of Birmingham Dubai, Dubai, United Arab Emirates

**Keywords:** Economic policy, Political economy, Users versus makers, Global North, Global South

## Abstract

The Global South and Global North terminologies, in addition to several other designations, have been used to classify the socio-economic nature of countries for centuries; however, these historical naming conventions carry subtle confusions and tend to tint political discourse. This research explores the classificatory differences in international trade, politics, economic theory, and the media and discusses how such differences inform the narratives surrounding world production and consumption systems, as well as trade. The current evidence suggests that several descriptions of the world economies create misunderstandings and often mischaracterize less developed countries while positively projecting certain countries as more advanced. I argue that, rather than using the current descriptions, the terms “users” and “makers” be adopted to characterize the nature of production and consumption in modern economies. The new terminologies are less arbitrary as they can eliminate the existing semantic problems commonly found in the media and within economic theory and political discourse. The innovative and simpler user/maker dichotomy provides a less prejudiced designation of nations and provides a new research dimension for political economy and management theory.

## Introduction

Some economists and political theorists might oppose a radicalist attempt to reframe existing economic development jargons into two forms, yet given that a dualist naming convention is less reckless and more mutualistic, its use might present a fairer perception of developing nations in particular. Far from being simplistic, the dualist characterization of economies and how global markets operate can be based on two economic operators: *users* and *makers* — each belonging to one end of a hypothetical continuum. From economic systems perspective, there will always be a *maker* — who creates the good or service, and the *user* — the one who utilizes or consumes the good or service. The view of economies operating as related parts of a system is derived from post-modern economic theory and is neither new nor radical. It forms the basis for the circular flow of income paradigm in macroeconomic theory and aids our understanding of equilibrium analysis in labor economics, for example, Mankiw and Taylor (2020). In this context, policy makers and theorist might perceive the new classification, proposed in this paper, as somehow less rejectionist (Chowdhury, 2021) and more an epistemological attempt that stimulates novel discourses.

Contrary to having less controversial definitions, textbooks and journals, daily business reports, and political news continue to refer to the world commonly and conveniently as being divided into confusing categories, for example, “developed” and “developing” countries or “First World” versus “Third World” (Cooke, 2004; Dellmuth & Schlipphak, 2020; Horner, 2020). These classifications have been extensively used by established international organizations (IMF, 2019; United Nations, 2019), governments, and the news media but also to a large extent by the academia (Bird et al*.*, 2016; Mensah, 2019) for several decades. Generally, we tend not to question the appropriateness of terms and concepts when their use have unconsciously solidify in the literature and taken for granted in popular use; so it is not surprising that although some terms remain widely vague and meaningless, their usage has continued to exist up to the twenty-first century. The classifications of the world into developed vs developing, for example, has been challenged earlier by Rodney (1972) and recently by several others (Madzík et al., 2015; Fialho & Van Bergeijk, 2017), yet, no plausible alternative classificatory hypothesis has been put forward. This study aims to analyze the shortcomings of popular phrases and terms used today and their damaging consequences on the perception and actions of individuals, businesses, governments, and international organizations who apply them in their research and decisions. A more direct outcome of this paper is to explore the feasibility of the novel classification “makers” vs “users” in an attempt to address the problems associated with the use of existing conventions, terms, and phrases.

## Methodology and Scope of the Literature

### Methodology

This is a review of existing taxonomies rather than regurgitation of generic literature in the field of development and economics. Given the nature of the study, two theoretical approaches informed the review — Glanzberg’s semantic theory and Cooper’s taxonomy: (1) Glanzberg (2009) proposes that “relativity of truth to a world plays no significant role in empirical semantic as empirical work in semantics is done not only against a background of the metatheory of formal languages, but also of philosophical ideas about content.” As such, this study evaluates existing classifications for content and semantics. (2) To collate and review the extant literature, Cooper’s (1988) taxonomy of literature review framework was applied whereby three out of the six designated criteria (that is, (a) focus, (b) goal, and (c) perspective) were applied. One key focus of this review is to layout the existing classifications used by the United Nations (UN), the International Monetary Fund (IMF), the World Bank (WB), and any other definitions to see areas of consistency and ambiguities. The goal, therefore, is to investigate if the current literature offers linguistic bridge-building, linguistic stereotyping/generalization, or polysemic conflict. Cooper’s perspective is addressed by providing an alternative proposition through a somewhat neutral representation of what could de-emphasize stereotypes and hegemony. The review was implemented for evidence of several traditional classifications frequently mentioned in economics, political, and social research.

### Traditional Dualist Classification of Economies

Given that the traditional classifications are often dualist in nature (Nielsen, 2013), it is confounding that recent classifications are pluralist in nature, for example, developing, developed, advanced, Third World, emerging markets, emerging economies, G7, G20, middle income (Amatori & Jones, 2003; Cuervo-Cazurra & Ramamurti, 2017). As the terminologies used in describing countries move away from what typically defines productive abilities, trade, and exchange, an opportunity exists for us to start rethinking what exactly it is that economies do — this should perhaps be the sole characterization of economies within business and economics discourse.

According to Table 1, traditional forms are less pluralistic and often, binary for almost all economies, for example, “developed” and “developing,” which is arguably one of the most commonly used taxonomies (Fialho & Van Bergeijk, 2017). A “developed country,” also known as “industrialized country,” is often defined as economies that achieve high levels of GDP per capita (Lambert D’raven & Pasha-Zaidi, 2016), market liberalization and political preferences (Grosjean & Senik, 2011), technology, and overall living standards (Madzík et al., 2015), while a “developing country” does not satisfy such standards. Despite this technical contrast, comparison among developing countries creates an obvious confusion as countries such as China, Namibia, Ukraine, Chile, and Samoa have vast differences among them, therefore making any such groupings technically erroneous. As a forced binary categorization, a large number of countries with wide-ranging levels of economic development are placed in the same group, which may limit the value of this division, as it is impossible to define shared characteristics of developing countries other than the fact that they are not considered “developed.” Despite the questions and confusions raised by writers like Rodney (1972), existing terminologies continued to be used by the International Monetary Fund (IMF) as can be seen in Table 1. Other international organizations, for example, the United Nations (UN, 2019) and World Bank (World Bank, 2019), acknowledge that a multitude of development levels exists globally and a significant number of countries cannot be classified as simply developed or developing yet; alternatives are hardly used in the politico-economic discourse.

**Table 1:** Division of economies in the IMF database

Country group	Notes
Advanced economies	Includes Euro area Major advanced economies (G7/G8)
Emerging market and developing economies	Emerging and developing Asia
	Emerging and developing Europe
	Latin America and the Caribbean
	Middle East and Central Asia
	Sub-Saharan Africa

Source: United Nations (2020)

### International Monetary Fund (IMF) Classification

The classification report published by the World Economic Outlook twice a year by the IMF offers the most comprehensive categorization of countries while providing advancement to the earlier dichotomized classification in the sense that it offers pluralistic categories among member countries (UN, 2019). While the “advanced economies” category is considered interchangeable with developed countries (UN, 2019), further subgroups are listed under this section: the euro area comprising 19 countries that use the euro as their official currency and major advanced economies elsewhere normally dubbed the G7. The G7 category comprises seven advanced economies with a relatively large population and sizable global economic influence (i.e., the USA, Japan, Germany, the UK, France, Italy, Canada); however, the G7 notation is semantically pompous and theoretically confusing — thus leading to the question, what exactly is *great* about these countries? Interestingly, Germany, France, and Italy appear in both subgroups, while some advanced economies are not listed in either subgroups (Australia, Czech Republic, Denmark, Hong Kong, Iceland, Israel, South Korea, Macau, New Zealand, Norway, Puerto Rico, San Marino, Singapore, Sweden, Switzerland, and Taiwan). Such apparent confusions were pointed out by Rodney (1972) several decades ago except that his focus at the time was quite Afrocentric and overly critical of Europe’s role in under-developing Africa. While acknowledging Rodney, it must be equally said that questions remain around the querulous approach taken in his book.

Another common grouping known as “emerging market” (Wafler & Badir, 2017; Jonnalagedda & Saranga, 2019) or “developing economies” (Fialho & Van Bergeijk, 2017; Thuy Hang Dao & von der Heidt, 2018) is often understood as another cliché for developing countries. A further subgrouping of 5 categories is provided based on geographic regions as “emerging and developing Asia,” “emerging and developing Europe,” “Latin America and the Caribbean,” “Middle East and Central Asia,” and “sub-Saharan Africa” — see Table 4.

A striking feature of the IMF classification is that dividing economies into two groups is not based entirely on GDP per capita, which is usually one of the most cited indicators used to differentiate rich countries from the poorer ones (Nielsen, 2013). For example, despite the fact that Macao SAR, Kuwait, and Brunei enjoy extremely high level of GDP per capita, they are not included in the list of advanced economies since the overwhelming majority of their income is from undiversified sources. An obvious question that arises is should economic development have to meet other criteria and, if so, what is it? Additionally, the lack of shared characteristics among developing countries defined by the IMF as the category includes various development levels (Rodney, 1972), ranging from the most fragile economies in sub-Saharan Africa to more prosperous ones in Eastern Europe (Thuy Hang Dao & von der Heidt, 2018), or from large- or medium-sized powerhouses such as Brazil and Indonesia to tiny island countries in the Pacific. A potential consequence of having such a broad category is that discussions about developing countries may fall into the trap of overgeneralizing (Fialho and Van Bergeijk, 2017), while policy makers may lack understanding of what the meaning and antecedents of sustainable development actually are (Mensah, 2019). For example, a trade agreement between the European Union (EU) and Brazil on sugar is unlikely to be identical to another agreement between the EU and Swaziland, which is a relatively small landlocked country in Southern Africa, even though both Brazil and Swaziland are considered developing economies by the IMF, hence calling into question what the purpose of the classification really is (Fialho & Van Bergeijk, 2017). As a consequence, analysis of global economic trends and policies designed to address international issues is subjected to gross assumptions, resulting in distorted perception and hegemonic privileges (Iyase & Folarin, 2018) for the architects of such divisive categorizations. On a similar critical note, De Beukelaer (2014) criticized the UNCTAD Creative Economy Reports, which claimed that the share of developing countries in the creative industry was almost equal to that of developed countries. The stunning performance of developing countries was explained by the presence of China in the developing category, while other developing countries individually performed rather badly. Similarly, the conclusion that global inequality has declined since the 1960s, propagated by high-profile publications (including those by the UN), was disproved by Hickel (2017), who argued that most of the convergence between the developed and developing world was mainly driven by growth in China. Pueyo and Linares (2013) suggested that attempts by the United Nations Framework Convention on Climate Change (UNFCCC) to transfer renewable technology to developing countries achieved limited success due to their homogeneous approach when dealing with developing economies. A concern that needs addressing in the literature is whether we have been complacent in the use of traditional stereotypic classifications to the extent that the lens used by the UN Security Council (Iyase & Folarin, 2018; Stephen, 2018), for example, can only see “developed” and “developing” as the prominent categories, despite such view limiting the understanding of current issues and the chances of providing effective solution in the future (Harris et al., 2009).

### United Nations (UN) Classification

There appears to be a notional similarity between how the IMF views the world as compared to how the UN who divides all economies into three groups: developed economies, economies in transition, and developing countries (The UN, 2019). Despite the addition of economies in transition to differentiate their classification from a binary one (see Table 3), it is not clear what such countries are transitioning to (Rodney, 1972) or how long such process will take (Dellmuth & Schlipphak, 2020). Further questions abound in terms of the legitimacy of the intermediate classification which would have been coined without consultation with countries that belong to this category (Stephen, 2018) rendering this classification extremely flawed and politically biased (Iyase & Folarin, 2018).

Attention should now be drawn to the specific issues that relate to the use of the IMF “economies in transition” conversion. (1) Why do they include countries of two regions: Southeastern Europe and former states within the Soviet Union instead of forming a novel group that comprises a narrow geographical region associated with the Cold War politics (Mastanduno, 2019)? (2) Why does the classification for “developed countries” include exclusively market economies of the West (with exception of Japan)? (3) Are communist, former Soviet Union, and Monarchical Nations strategically excluded from developed nations despite having either completed their transformation to full-fledged market economies or showing huge signs of growth, stability, and happiness? For example Poland, Slovenia, and Bulgaria on one hand and the United Arab Emirates, Singapore, and Qatar have very high GDP per capita and could easily be classified as developed. It is quite possible that the newly introduced grouping was created based on a Eurocentric political agenda that overemphasizes links to historical events in Europe and indicates little consideration for development in other parts of the world (Korukonda, 2007; Norrlof & Wohlforth, 2019). The classification may imply that only countries in Central and Eastern Europe can progress to become developed economies while countries in Asia, Africa, and Latin America remain static in the lowest tier.

We can safely infer suspicion as to why the UN classification completely disregards actual economic performance of non-Western economies in recent decades, resulting in inconsistent and unrealistic categorization (Fialho & Van Bergeijk, 2017; Cooley, 2019; Mensah, 2019). For instance, it is questionable that Hong Kong, Singapore, South Korea, and Taiwan, 4 of the most technologically advanced and prosperous economies in Asia, are still placed in the developing category, while former Soviet countries with much lower GDP per capita such as Uzbekistan and Moldova are considered transitioning economies.

Besides the simplistic division of economies into 3 vaguely defined categories (Table 3), the UN classification also includes several additional categories such as economies by per capita gross national income (GNI), least developed countries, heavily indebted poor countries, small island developing states, and landlocked developing countries (UN, 2019). Among these groups, the classification based on per capita GNI resembles the WB classification in Table 3 with some slight variance in the number of countries included.

### The World Bank (WB) Classification

The World Bank classifies economies into four main groups based on GNI (nominal) per capita: high income, upper middle income, lower middle income and low income (World Bank, 2019). While the category of high-income countries in Table 5 largely corresponds to “developed countries” in Table 2, it is not clear what would be the matching a criterion for emerging countries on economies in transition mentioned in other classifications. Despite that, the thresholds of each income group are revised by the WB every year making their criteria more progressive and largely more realistically close to the living standards definitions (Madzík et al., 2015), except that the parameters are still determined by dominant nations (Stephen, 2018).

**Table 2:** List of developed economies (United Nations, 2019)

North America	Europe		Major developed economies (G7)
	European Union	Other Europe	
Canada	**EU-15**	Iceland	Canada
USA	Austria ^a^	Norway	Japan
	Belgium ^a^	Switzerland	France
	Denmark		Germany
	Finland ^a^		Italy
	France ^a^		UK
	Germany ^a^		USA
	Greece ^a^		
	Ireland ^a^		
	Italy ^a^		
	Luxembourg ^a^		
	Netherlands ^a^		
	Portugal ^a^		
	Spain ^a^		
	Sweden		
	UK ^b^		
Developed Asia and Pacific	**EU-13** ^c^		
Australia	Bulgaria		
Japan	Croatia		
New Zealand	Cyprus ^a^		
	Czech Republic		
	Estonia ^a^		
	Hungary		
	Latvia ^a^		
	Lithuania ^a^		
	Malta ^a^		
	Poland		
	Romania		
	Slovakia ^a^		
	Slovenia ^a^		

Source: United Nations (2019)

Superscripts exempted for this study

**Table 3:** List of economies in transition (United Nations, 2019)

Southeastern Europe	Commonwealth of independent States and Georgia ^a^	
Albania	Armenia	Republic of Moldova
Bosnia and Herzegovina	Azerbaijan	Russian Federation
Montenegro	Belarus	Tajikistan
Serbia	Georgia ^a^	Turkmenistan
The Former Yugoslav Republic of Macedonia	Kazakhstan	Ukraine ^b^
	Kyrgyzstan	Uzbekistan

Source: United Nations (2019)

Superscripts exempted for this study

**Table 4:** Developing Economies by region

	Africa	Asia	Latin America and the Caribbean
North Africa	South Africa	East Asia^b^	Caribbean
Algeria	Angola	Brunei Darussalam	Bahamas
Egypt	Botswana	Cambodia	Barbados
Libya	Eswatini	China	Belize
Mauritania	Lesotho	Democratic People’s Republic of Korea^c^	Guyana
Morocco	Malawi		Jamaica
Sudan	Mauritius	Fiji	Suriname
Tunisia	Mozambique	Hong Kong SAR^d^	Trinidad and Tobago
**Central Africa**	Namibia	Indonesia	**Mexico and Central America**
Cameroon	South Africa	Kiribati	Costa Rica
Central Africa Republic	Zambia	Lao People’s Democratic Republic	Cuba
	Zimbabwe		Dominican Republic
Chad	**West Africa**	Malaysia	El Salvador
Congo	Benin	Mongolia	Guatemala
Equatorial Guinea	Burkina	Myanmar	Haiti
Gabon	Cabo Verde	Papua New Guinea	Honduras
Sao Tome and Principe	Cote d’Ivoire	Philippines	Mexico
**East Africa**	Gambia (Islamic Republic of the Gambia)	Republic of Korea	Nicaragua
Burundi		Samoa	Panama
Comoros	Ghana	Singapore	**South America**
Democratic Republic of the Congo	Guinea	Solomon islands	Argentina
	Guinea-Bissau	Taiwan Province of China	Bolivia (Plurinational State of Bolivia)
Djibouti	Liberia	Thailand	
Eritrea	Mali	Timor-Leste	Brazil
Ethiopia	Niger	Vanuatu	Chile
Kenya	Nigeria	Vietnam	Colombia
Madagascar	Senegal	**South Asia**	Ecuador
Rwanda	Sierra Leone	Afghanistan	Paraguay
Somalia	Togo	Bangladesh	Peru
South Sudan^c^		Bhutan	Uruguay
Uganda		India	Venezuela (Bolivarian Republic of Venezuela)
United Republic of Tanzania		Iran (Islamic Republic of Iran)	
		Maldives	
		Nepal	
		Pakistan	
		Sri Lanka	
		**Western Asia**	
		Bahrain	
		Iraq	
		Israel	
		Jordan	
		Kuwait	
		Lebanon	
		Oman	
		Qatar	
		Saudi Arabia	
		State of Palestine^c^	
		Syrian Arab Republic	
		Turkey	
		United Arab Emirates	
		Yemen	

Source: United Nations (2019)

Superscripts exempted for this study

**Table 5:** Truncated World Bank list of countries by income level

x	x	Economy	Code	X	Region	Income group	Lending category	Other
x	x	x	x	x	x	x	x	x
1		Afghanistan	AFG		South Asia	Low income	IDA	HIPC
2		Albania	ALB		Europe & Central Asia	Upper middle income	IBRD	
3		Algeria	**DZA**		Middle East & North Africa	**Lower middle income**	IBRD	
4		American Samoa	ASM		East Asia & Pacific	Upper middle income	..	
5		Andorra	AND		Europe & Central Asia	High income	..	
6		Angola	AGO		Sub-Saharan Africa	Lower middle income	IBRD	
7		Antigua and Barbuda	ATG		Latin America & Caribbean	High income	IBRD	
8		Argentina	ARG		Latin America & Caribbean	Upper middle income	IBRD	
9		Armenia	ARM		Europe & Central Asia	Upper middle income	IBRD	
10		Aruba	ABW		Latin America & Caribbean	High income	..	
11		Australia	AUS		East Asia & Pacific	High income	..	
12		Austria	AUT		Europe & Central Asia	High income	..	EMU
13		Azerbaijan	AZE		Europe & Central Asia	Upper middle income	IBRD	
14		Bahamas, The	BHS		Latin America & Caribbean	High income	..	
15		Bahrain	BHR		Middle East & North Africa	High income	..	
16		Bangladesh	BGD		South Asia	Lower middle income	IDA	
17		Barbados	BRB		Latin America & Caribbean	High income	..	
18		Belarus	BLR		Europe & Central Asia	Upper middle income	IBRD	
19		Belgium	BEL		Europe & Central Asia	High income	..	EMU
20		Belize	BLZ		Latin America & Caribbean	Upper middle income	IBRD	
21		Benin	**BEN**		Sub-Saharan Africa	**Lower middle income**	IDA	HIPC
22		Bermuda	BMU		North America	High income	..	
23		Bhutan	BTN		South Asia	Lower middle income	IDA	
24		Bolivia	BOL		Latin America & Caribbean	Lower middle income	IBRD	HIPC
25		Bosnia and Herzegovina	BIH		Europe & Central Asia	Upper middle income	IBRD	
26		Botswana	BWA		Sub-Saharan Africa	Upper middle income	IBRD	
27		Brazil	BRA		Latin America & Caribbean	Upper middle income	IBRD	
28		British Virgin Islands	VGB		Latin America & Caribbean	High income	..	
29		Brunei Darussalam	BRN		East Asia & Pacific	High income	..	
30		Bulgaria	BGR		Europe & Central Asia	Upper middle income	IBRD	
31		Burkina Faso	BFA		Sub-Saharan Africa	Low income	IDA	HIPC
32		Burundi	BDI		Sub-Saharan Africa	Low income	IDA	HIPC

Source: World Bank (2019)

(1) Bold indicates a change of classification. (2) Economies are divided among income groups according to 2019 gross national income (GNI) per capita, calculated using the World Bank Atlas method. The groups are low income, $1035 or less; lower middle income, $1036–4045; upper middle income, $4046–12,535; and high income, $12,536 or more. The effective operational cutoff for IDA eligibility is $1185 or less. (3) Data extracted from a group of 189 member countries

**Table 6:** Summary of classificatory issues

Terminology	Ambiguous	Stereotypical/derogatory	Hegemonic	Economic (**E**)/political (**P**)	Contemporary relevance
Developed	✓			E, P	✓
Developing	✓	✓	✓	E, P	
Under-developed		✓	✓	P	
Economies in transition	✓	✓	✓	E, P	
The West	✓			P	✓
The East	✓	✓	✓	P	✓
Modern world	✓			P	
First World				P	
Third World		✓	✓	P	
Global North	✓			P	
Global South	✓	✓	✓	P	

As a more realistic taxonomy, the WB classification into four groupings helps assuage the issue of excessively broad spectrum of “developing countries.” While according to the IMF classification, Colombia, the Philippines, and Togo are all considered developing countries, the WB method assigns each of these countries to three distinct groups: upper middle income (Colombia), lower middle income (Philippines), and low income (Togo). Therefore, these terms allow both economists and the public to describe and compare development level of economies with higher accuracy than other parameters.

Still, some writers (Stephen, 2018; Dellmuth & Schlipphak, 2020) argue that the classification of the WB can still be further improved for several reasons. First, the use of only one indicator (GNI per capita) can possibly lead to inaccurate classification of economies. For instance, both Brunei and Singapore are classified as high-income countries though their economic structures are vastly divergent; while the former is reliant on export of a single commodity (Anaman, 2004), the latter possesses a diversified economy driven by entrepreneurship and technology (Austin, 2009; Peng & Phang, 2018). This confusion leads to legitimately questioning the validity of using only GNI per capita to compare countries while leaving questions of inequality within the country unanswered. Among African countries, South Africa is ranked a higher-middle-income country, although its citizens experience the highest level of unequal wealth distribution, resulting in significantly lower performance on educational and health indicators compared to countries of similar income level. Second, it is doubtful if the WB’s four-level categorization can be perceived as better than earlier binary classifications; if the assumption is true, then there might need to be further sub-categorizations in future that might pose further conceptual ambiguities. Vázquez and Sumner (2013) argued that for developing countries exclusively, five clusters are required to describe their development patterns. Third, there is a potential contention that the addition of upper-middle-income and lower-middle-income categories will not fully address problems generated by considering countries as single economic units. Sumner (2010) pointed out that today, the majority of poor people do not reside in least developed or low-income countries: in 2007–2008, low-income countries accounted for only one quarter of the global poor, while the other three quarters lived in middle-income countries. This can be explained by the reclassification of heavily populated countries (India, Indonesia, and the Philippines) from low to middle-income levels based on average national statistics despite the fact that millions of residents in these countries did not escape poverty.

### Other Politico-economic Classifications

#### First World vs Third World

A contrast that is often used to separate developed countries from developing economies is the “First World” and “Third World” terminology. However, the absence of the “Second World” in this classification often leads to confusion, while some have questioned the appropriateness of the term (Weinhardt, 2020) and the terms limited opportunities for theoretical advancement (Dellmuth & Schlipphak, 2020). At first, the negative connotation of the term Third World can be taken for granted, but its specific use in some cases can have a direct negative implication as in the case of HIV studies (Bird et al., 2016) and in the study of wars and politics (Wade, 2011). Like most terminologies that have been coined several decades ago, the above terminologies used in describing economies and its people are seemingly attracting criticism due to their obsolescence.

Politico-economic terms have the tendency of being linked to colonial era (Ameyaw Domfeh, 2004) or the cold war era (Cooley, 2019; Mastanduno, 2019). Between the late 1940s and early 1950s, the tripartite division of the world emerged as the USA and Soviet Union advocated for opposite economic models and political systems (Cooley, 2019). However, a large number of African and Asian countries although gaining independence or in the process of gaining independence were reluctant to completely align with one of the two superpowers (Berger, 2004). In the 1975 version of the three-world model, the First World comprised the USA and its allies in Western Europe, Australia, and Japan; the Second World included communist countries that followed a centrally planned economy such as the Soviet Union, Eastern European countries, Cuba, China, North Korea, and Vietnam; and the Third World encompassed all remaining countries that were not classified in either of the two groups. As tension between the two superpowers escalated between the 1950s and 1970s, many newly independent countries in Asia and Africa were unwilling to align with either the First or Second Worlds and attempted to form a new group, culminating in the Bandung Conference in 1955 and the establishment of the Non-Aligned Movement. However, since most of the non-aligned countries are developing economies, the term “Third World” has been often associated with lack of development or underdevelopment (Kamrava, 1993). The use of this classification is now common in the academic literature (Sunwolf, 2006; Peterson, 2012; Weinhardt, 2020), thereby providing journalists and reporters opportunity to freely use the terms with little caution. The collapse of the Soviet Union and the centrally planned economic model in the vast majority of former communist countries (Mastanduno, 2019) has resulted in the disappearance of the Second World and the consequential reclassification of these economies. For example, the World Bank due to their rapid transition to market economy has upgraded the Czech Republic and Latvia to the high-income tier with rising living standards; thus, these economies can now be considered First World by the stretch of the classification. Despite such a progress, some might see placing fast-growing economies such as China and Vietnam into the Third World category as rather controversial because of the outdated nature of the term akin to the developing counties classification. Today, it can be argued that the Third World is no more than a vague list of non-First World countries that share little in common purely based on an unrealistic and discriminatory agenda. Shu-Yun (1998) suggested that the disappearance of the Second World renders the spirit of non-alignment of the Third World irrelevant, leading potentially to the decline of Third World studies. Randall (2004) claimed that the notion of the Third World is no longer useful as specialist knowledge about individual economies as these regions grow, demonstrating increasing differentiation among previously classified nations.

#### The West vs the East

A common identifier used by a vast majority of writers in the media and in the academic sector is the “West and East” divide. Although geographically appropriate (Hettne, 2005) and perfectly valid in other fields, the political application of the term and its popular use in policy development can be problematic (Shu-Yun, 1998). While the “West” classification usually comprises North America, Europe, Australia, New Zealand, and possibly Latin America, the East refers to countries in Asia, sometimes referred to as the Far East; however, this is where the semantic confusion starts. In politics and seldom in economics discourse, it is not clear where the dividing line is between the East and the West and where nations of Africa belong in this rather anachronistic divide. The rise of the West–East dichotomy is possibly a result of both the traditional Eurocentric attitude and competition for global power (Porter, 2019) and politico-economic hegemony (Gause, 2019; Ikenberry and Nexon, 2019). In recent times, the massive economic development in Asia (Peng & Phang, 2018; de Graaff et al., 2020) has rendered the pejorative meaning of the term East in the historic literature untenable and meaningless. By 2019, nominal GDP of China, Japan, and India surpassed that of the UK, France, and Italy (UN, 2019). Four Asian tigers (Singapore, Hong Kong, South Korea, and Taiwan) achieved higher GDP per capita compared to several Southern European countries. Strikingly, among the developed members of the Eastern world, Singapore and Vietnam have recorded enviable growth rates by the year 2010 (Hanh et al., 2017; Peng & Phang, 2018), while some traditionally powerful economies have stalled in terms of GDP growth. In other words, countries in the East are slowly becoming significant counterweight to the economic powers of the West and thereby potentially balancing the economic power of the world (Cooley, 2019).

Turning now to the exclusion of countries that do not perfectly fall into East/West divide, we find that a semantic discrimination exists; that is, if a country is neither part of the East nor the West, then what will they be? The Middle East, which is located between the West and the East, is possibly overlooked even though countries such as Turkey, the UAE, and Egypt have gained significant economic influence in the region and become important trading partners with various economies in both the West and the East, while Africa is given no prominence. With a population of over 1.2 billion people and more than 50 countries, it is both ethically wrong and academically careless to excluded Africa from this classification while potentially mis-defining the geography (Culcasi, 2010) and the economic relevance of the region.

#### Global North vs Global South

The immediate concern posed by “Global North” vs “Global South” classification is that it has the tendency of contradicting the earlier East/West classification. This fairly new classification was popularized in the second half of the twentieth century and continues to be used in economic and political discussion (Horner, 2020) while serving as basis for cooperation on a global scale among developing countries such as South–South Cooperation and Group of 77 (G77). While the North represents North America, Europe (including several former Soviet Union states such as Russia and Kazakhstan), Turkey, Japan, South Korea, Taiwan, Singapore, Australia, and New Zealand, the South broadly represents all other countries. While not exclusively based on geography, the terminology conflicts with the earlier offered version of developed and developing version of countries as the Global South tend to be less economically successful and competitive. To Global North countries, the Global South has become a convenient term to describe a world of mostly non-European, post-colonial nations that lack economic opportunities and largely hampered by corrupt governance (Comaroff & Comaroff, 2012).

Like earlier versions, a number of arguments challenge the division of the world into two separate categories — *North* and *South*. Firstly, this simplistic approach ignores the vast divergence of development within each category of the grouping. Not all countries in the Global South experience abject poverty, for instance, Uruguay, Botswana, and Malaysia, all belong to the South group, despite having successfully attained high levels of human development. Secondly, this dichotomy can be criticized as stereotypical and discriminatory against countries defined as the South. Ratuva (2016) claimed that the “West versus the rest’ mentality of mainstream Western discourses leads to a flawed perception in which the South is abundant of security threats to the value of the West. In reality, several regions of the Global North also face socio-economic and political challenges such as high unemployment rates in Spain and Italy (Verd et al., 2019). Meanwhile, evidence suggests that countries in Africa (Fialho & Van Bergeijk, 2017) and indeed Asia are developing significantly to avoid being categorized as poor (Anaman, 2004; Peng & Phang, 2018). This point is further strengthen by the increasing standards of living in Shanghai (China) currently comparable to cities in the North, while Costa Rica in Central America champions efforts in sustainable development based on a diversified economy and strong regulations to preserve the environment (Herrero Amo & De Stefano, 2019).

One logical conclusion in this study is that the existing classifications, definitions, and taxonomies are not without flaws; to put the debate in context, a summary of terminologies with their associated issues is presented in Table 6.

## Alternative Classification of Economies and Propositions

### The Case for an Alternative Classification

**Fig. 1:**
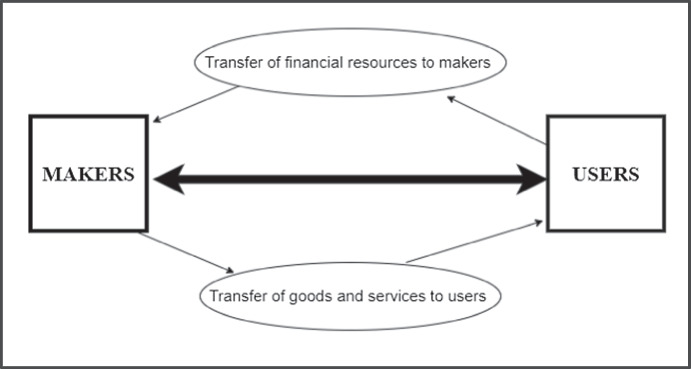
Model of interaction between makers and users. Alt text for the figure [15 words]: makers produce goods and services and transfer them to users in exchange for financial resources

It has been established repeatedly in this study that existing classifications present challenges to effective academic discourse (see Tables 5 and 6) and policymaking. This is compounded by the recent attack on political incorrectness (Dzenis and Nobre Faria, 2019), mainly in the media and politics, but particularly, in the academia where the phenomenon is under-studied (Maranto, 2020). A theoretically harmless alternative labeled “makers” and “users” is proposed in line with Glanzberg’s (2009) recommendation for semantic context, whereby makers are seen as economies that are capable of producing a surplus of goods and services (Mankiw & Taylor, 2020), while users are economies that consume more goods and services than they produce. Economies that make more than they use in the long term are able to accumulate more wealth, reflected in higher level of personal, business, and government savings; these financial resources can be reinvested in productive sectors of the economy to generate jobs while spending on human development goals (Mensah, 2019). In contrast, those that continuously use more goods and services than they make are likely to face economic challenges, including high inflation rates, dwindling foreign exchange reserves, and significant indebtedness (Mankiw & Taylor, 2020). While this latter point is a domain studied by macroeconomics, it is not necessarily taken for granted in this study, although the idea here is to construct a language that views users as partners in global economic exchange. As such, users tend to provide the (supplementary) financial resources needed by makers (for example, China and Germany) to support their production efforts. Makers provide the manufactured goods and services in exchange for financial resources from the users as shown in Fig. 1.

For the interactive system to work effectively in Fig. 1, we can assume that maker economies comprise those that possess advanced technology (particularly in several key industries such as IT, financial services, and education), modern infrastructure, well-established, and continuously innovative industrial base. Such factors allow the labor force of maker economies to produce high value goods and services desired by both citizens residing inside and outside the boundaries of makers: hypothetical examples of maker countries are Germany, South Korea, the UAE, and Taiwan. In contrast, economies in which a high percentage of working adults are employed in subsistence agriculture are less likely to produce goods and services desired by a large number of customers worldwide. Simultaneously, such economies may still consume a significant amount of goods and services provided by maker economies and foreign donors, leading to budget deficit or dependence on foreign aid.

According to Table 6, several classifications appear to be stereotypical and hegemonic in nature owing perhaps, to the overdependence of user countries on aids and grants that have historically been preferred by countries such as Ghana, Uganda, and Vietnam. Such overdependence lead to the WB classification of a number of countries as heavily indebted poor countries (HIPC) — see Table 5. While HIPC can attract unfavorable terms of trade (Hall, Karadas and Schlosky, 2018), at the economic front, it is crucial to note that export revenues only relate to the value of goods and services produced within the economy and consumed in other geographical areas and therefore do not include the value of products made and used by people within the economy itself. The maker–user framework considers production and consumption beyond the export–import framework as would be the case for a closed economy, for example.

The urgency for the adoption of the maker–user framework exists due to the poor use of sovereign nations as the basis for groupings. Using sovereign countries as primary units to classify economies in several circumstances creates confusion since this traditional approach is subject to considerable generalization. The problem arises as some national economies are vastly diverse and economic composition varies among subnational administrative divisions. For example, despite the fact that China is considered a manufacturing hub of the world and its national economy is likely to be a maker, this categorization may not necessarily apply to landlocked units of the country such as Yunnan or Qinghai. Thus, economies of subnational administrative divisions such as provinces or states should also be considered within the user–maker framework.

### Propositions

The existing traditional taxonomies have so far failed to undo the semantic confusions in the literature but further help solidify biased view and stereotypes of several fairly successful economies (Bird et al., 2016). Despite critics producing enviable accounts of how questionable current classifications are (Horner, 2020; Maranto, 2020), very little has been done to provide alternative definitions or classifications that are devoid of hegemonic intentions or stereotypes; hence:

- *Proposition 1:* When referring to economies that engage in significantly large amounts of production of services and goods, it is better to use the term “makers.” A maker will create value by providing goods and services that are used by citizens within the country as well as by other economies that are not able to produce such goods or have elected not to do so because of the dictates of adverse comparative advantage. By 2020, China, India, and Singapore have gained prominence in the creation of goods and services therefore could be safely classified as makers, ceteris paribus.
- *Proposition 2*: the term “users” refer to economies that utilize resources, products, and services created by makers. A user is able to create goods and services but maybe unable to do so due to steep cost curves or lack of comparative advantage. If the volume of goods and services purchased from other economies is proportionately high, then that country is a user.
- *Proposition 3*: from P1 and P2, it follows that for an economy to be deemed a maker or a user, one or both of the following criteria should apply. (1) Net export (which is the difference between a country’s export and import) must be positive for a maker and negative for a user, all other things being equal. (2) Despite trade deficits or balances, an economy will be a maker if a large volume of manufacturing and services is created for domestic use irrespective of whether these products form the basis for international trade or not and vice versa.
- *Proposition 4*: due to the potential confusion it brings when discussing economies of the world, the terms developed as opposed to developing or underdeveloped should be used with caution. The proposition applies to all terms currently deemed as hegemonic or stereotypical — see Table 6. For theoretical purposes, the more relevant classification makers vs users should be applied when making comparison among economies of the world.

## Implications for Practice and Research

The first implication for classifying economies into makers and users is that the relationship between production and consumption becomes the primary focus, instead of gross domestic product or gross national income. As a result, this classification serves a unique purpose in evaluating potential economic development of various economies as well as their long-term growth synergies.

There is a further implication for utilizing the new classification as the foundation for developing a better framework to study economies and related events while being able to recommend efficient policies in addressing challenges that hamper investigations in the field of economic development.

Lastly, avoiding stereotypes, stigmas, and hegemony in economic discourse is an effective approach to dealing with current global issues. As production and consumption choices and systems continue to change and recently become unpredictable (due to COVID-19), the language choices are even more important than before; hence, the current study plays a key role in defining how economies should be referred to.

## Limitations

As is the case with most critical research, this study is not without limitations. Firstly, the study only reviewed a selected number of terms and classifications; as such, it cannot be claimed that the criticisms laid out are adequately reflective of the general literature. Secondly, the proposition to classify economies as either a maker or user is a quantum leap that requires additional evidence and justification to give it prominence in the economic and political literature. In reality, several economies transition between user and maker status as they run out of resources or when the structure of the economy changes. Finally, a more reliable criteria by which a maker economy as opposed to a user economy can be determined is necessary; generally, economies can be both users and makers at the same time so the proposition (in Fig. 1) that economies be classified as either might not always hold.

## Conclusion

Notwithstanding the semantic constraints and logical confusions that impact the meaning and usage of terms used in classifying economies, this study has demonstrated the weaknesses of popular classifications of economies, which often result in flawed insights and inefficient strategies resolving global challenges. Previous classifications either vaguely grouped a large number of economies with relatively partial resemblance into a single category while describing non-Western, non-developed economies with questionable, discriminatory, and stereotypical language. In response to these issues, this study advocates for a new type of classification based on actual production and consumption of goods and services within an economy. Further research is crucial to improve the validity and reliability of preliminary work so that accurate description of the maker and user economies can be provided.
